# Machine learning can predict mild cognitive impairment in Parkinson's disease

**DOI:** 10.3389/fneur.2022.1010147

**Published:** 2022-11-17

**Authors:** Marianna Amboni, Carlo Ricciardi, Sarah Adamo, Emanuele Nicolai, Antonio Volzone, Roberto Erro, Sofia Cuoco, Giuseppe Cesarelli, Luca Basso, Giovanni D'Addio, Marco Salvatore, Leonardo Pace, Paolo Barone

**Affiliations:** ^1^Department of Medicine, Surgery and Dentistry “Scuola Medica Salernitana”, University of Salerno, Baronissi, Italy; ^2^IDC Hermitage-Capodimonte, Naples, Italy; ^3^Department of Electrical Engineering and Information Technologies, University of Naples “Federico II”, Naples, Italy; ^4^Bioengineering Unit, Institute of Care and Scientific Research Maugeri, Telese Terme, Italy; ^5^IRCCS SDN SYNLAB, Naples, Italy; ^6^Department of Chemical, Materials and Production Engineering, University of Naples “Federico II”, Naples, Italy

**Keywords:** mild cognitive impairment, machine learning, gait analysis, amyloid PET imaging, Parkinson's disease

## Abstract

**Background:**

Clinical markers of cognitive decline in Parkinson's disease (PD) encompass several mental non-motor symptoms such as hallucinations, apathy, anxiety, and depression. Furthermore, freezing of gait (FOG) and specific gait alterations have been associated with cognitive dysfunction in PD. Finally, although low cerebrospinal fluid levels of amyloid-β42 have been found to predict cognitive decline in PD, hitherto PET imaging of amyloid-β (Aβ) failed to consistently demonstrate the association between Aβ plaques deposition and mild cognitive impairment in PD (PD-MCI).

**Aim:**

Finding significant features associated with PD-MCI through a machine learning approach.

**Patients and methods:**

Patients were assessed with an extensive clinical and neuropsychological examination. Clinical evaluation included the assessment of mental non-motor symptoms and FOG using the specific items of the MDS-UPDRS I and II. Based on the neuropsychological examination, patients were classified as subjects without and with MCI (noPD-MCI, PD-MCI). All patients were evaluated using a motion analysis system. A subgroup of PD patients also underwent amyloid PET imaging. PD-MCI and noPD-MCI subjects were compared with a univariate statistical analysis on demographic data, clinical features, gait analysis variables, and amyloid PET data. Then, machine learning analysis was performed two times: Model 1 was implemented with age, clinical variables (hallucinations/psychosis, depression, anxiety, apathy, sleep problems, FOG), and gait features, while Model 2, including only the subgroup performing PET, was implemented with PET variables combined with the top five features of the former model.

**Results:**

Seventy-five PD patients were enrolled (33 PD-MCI and 42 noPD-MCI). PD-MCI vs. noPD-MCI resulted in older and showed worse gait patterns, mainly characterized by increased dynamic instability and reduced step length; when comparing amyloid PET data, the two groups did not differ. Regarding the machine learning analyses, evaluation metrics were satisfactory for Model 1 overcoming 80% for accuracy and specificity, whereas they were disappointing for Model 2.

**Conclusions:**

This study demonstrates that machine learning implemented with specific clinical features and gait variables exhibits high accuracy in predicting PD-MCI, whereas amyloid PET imaging is not able to increase prediction. Additionally, our results prompt that a data mining approach on certain gait parameters might represent a reliable surrogate biomarker of PD-MCI.

## Introduction

Over the past few years, research has focused on the pre-dementia stage of cognitive impairment in Parkinson's Disease (PD), namely mild cognitive impairment (PD-MCI). PD-MCI has a pooled prevalence of 40% (1) and is a risk factor for the development of dementia associated with PD (PDD) (2, 3). Several mechanisms are likely to contribute to cognitive decline in PD (4).

Clinical markers of cognitive dysfunction in PD encompass several mental non-motor symptoms such as visual hallucinations, apathy, anxiety, and depression (1, 5, 6). Besides mental symptoms, a growing body of evidence indicates the association between cognitive decline and several gait and balance dysfunction, including the postural instability gait difficulty (PIGD) phenotype (7, 8), freezing of gait (FOG) (9–11), and specific walking alterations, especially in dual-task conditions, on quantitative gait evaluations (12–16). In addition, although the evidence consistently suggests that low cerebrospinal fluid levels of amyloid-β42, a marker of Alzheimer's disease (AD), may predict future cognitive decline and PDD (5), to date, amyloid-β (Aβ) Positron Emission Tomography (PET) imaging studies have reported conflicting results (17–21), thus failing to consistently demonstrate an association between Aβ plaques deposition and cognitive dysfunction in PD. Of note, many cross-sectional studies showed a relationship between Aβ pathology and the PIGD subtype in PD (22–25). These findings may suggest that both cognition and gait dysfunction in PD might share common underlying pathological mechanisms which, at least in part, could be related to Aβ pathology.

Since PDD is associated with greater disability, caregiver burden, and risk for institutionalization with an increase in health-related costs (26), the identification of factors and biomarkers associated with MCI might allow the early detection of a PD subpopulation at a higher risk of worse disease progression (27). Form a research prospective, detecting such patients and their course will foster the development of effective strategies to prevent or delay progression to dementia.

In the recent past, the application of machine learning to medical datasets has increased. Machine learning techniques are computer-based statistical approaches, which can be trained to find common patterns and hidden correlations from big amounts of data. Indeed, machine learning methods can help clinicians in classifying at once patients according to several variables (28).

The main aim of the present study was 2-fold: (1) identifying the top features associated with PD-MCI among those commonly reported in previous research; and (2) detecting a machine learning algorithm able to distinguish PD patients with MCI from those without. To this objective, five machine learning algorithms, namely Random Forest (RF), Support Vector Machine (SVM), k-Nearest Neighbor (KNN), Decision Tree (J48), and AdaBoost (ADA-B), were implemented using a multiparametric approach including clinical features generally associated with cognitive decline in PD, gait parameters under dual-task condition obtained through gait analysis, and amyloid PET imaging data.

## Materials and methods

### Study design and population

The study population consisted of 75 PD patients, consecutively enrolled between February 2018 and November 2021. Participants were selected from patients referred to the Movement Disorders Unit of the Institute for Diagnosis and Care Hermitage-Capodimonte of Naples and the Center for Neurodegenerative Diseases of the University of Salerno. All patients fulfilled the Movement Disorder Society (MDS) clinical diagnostic criteria for PD (29). The inclusion criteria were as follows: age 45 years or older; Hoehn & Yahr (H&Y) score ≤3; disease duration <10 years; and anti-parkinsonian treatment at a stable dosage during the previous 4 weeks. The exclusion criteria were as follows: gait requiring assistance; dementia according to the DSM-V criteria; clinically significant comorbidities, including other neurologic disorders, orthopedic diseases, or cardiovascular/respiratory diseases; anticholinergic or neuroleptic treatment; and brain surgery.

This study was performed in accordance with the 1964 Declaration of Helsinki and was approved by the IRCCS G. Pascale Foundation, the reference ethics committee of the IRCCS SDN SYNLAB, Naples. Written informed consent was obtained from all subjects.

### Clinical and cognitive evaluations

Patients were assessed using an extensive clinical and neuropsychological examination. Clinical evaluation included the use of the Movement Disorder Society-Unified Parkinson's Disease Rating Scale (MDS-UPDRS) which was developed to evaluate various aspects of Parkinson's disease including non-motor and motor experiences of daily living and motor complications. The presence and severity of hallucinations/psychosis, apathy, sleep disorders, anxiety, depression (range 0–4), and the presence of FOG were rated using the specific items of the MDS-UPDRS I and II.

The cognitive assessment included an extensive neuropsychological battery comprising at least two tests for each of the five cognitive domains commonly affected in PD, namely memory, attention, executive function, visuospatial skills, and language. The test scores were corrected for current normative values. All neuropsychological tests were administered to the patients during the pharmacological on-state. Based on the neuropsychological examination, patients were classified as patients without (no PD-MCI, *n* = 42) and with MCI (PD-MCI, *n* = 33), according to level II criteria for PD-MCI (30).

### Gait analysis

All patients performed gait analysis through the optical motion capture system of BTS Bioengineering SMART DX, fitted out with six infrared cameras, two video cameras, two force plates, a set of passive markers, and an elaborator. A specific procedure was required for calibrating the volume of acquisition. Then, the Davis-Heel Protocol was applied for each acquisition (31), consisting of four sequential phases: anthropometric measures (height, weight, leg length, etc.); positioning of 22 reflective silver-covered markers on specific points on the body; standing phase, that is, acquiring the patient standing up on the force plate; and walking phase on a 10-m path. Patients were evaluated on the straight pathway during a dual task, namely walking while serial subtracting 7s starting from 100 (COG task), performed four times. Before starting the trial, no instruction to prioritize walking or calculating was given to all participants, who were trained to walk at a normal pace and at their usual speed. Through this gait analysis procedure, a final report was obtained with spatial and temporal parameters, all reported in **Table 2**.

All participants were assessed in the self-defined best “on-state” while receiving their typical dopaminergic drugs, based on previous findings showing that gait during the on-state rather than the off-state exhibits a closer connection with cognitive dysfunction (10, 12–14).

### Amyloid PET imaging

A subgroup of PD patients also underwent amyloid PET imaging. PET scans were acquired 90+5 min after [18F]flutemetamol i.v. administration (range 180–190 MBq) for 20 min in all subjects using a commercial scanner (Discovery PET/CT 710, GE Healthcare, Milwaukee, WI, USA) in 3D scanning mode that examined 47 slices of 3.3-mm thickness spanning the entire brain. Images were reconstructed using a 3D iterative ordered-subset expectation maximization (OSEM) and Time of Flight (TOF) technologies (GE VUE Point FX with SharpIR, GE Healthcare). The OSEM algorithm was applied to ratio sinograms using attenuation-weighted iterative reconstruction (7 iterations, 36 subsets) and all reconstructions always included a “weak” z-axis filter. Pet scans were interpreted using the approved image training instructions provided by the manufacturer (32). For quantitative analysis, the patient's brain was divided into 67 regions using the Automated Anatomical Labeling-Merged atlas with the software PMOD (PMOD Technologies, Zurich, Switzerland). Averaged Standardized Uptake Values (SUVs), defined as the decay corrected brain radioactivity concentration, normalized for injected dose and body weight, were calculated for all regions and used to evaluate tracer retention in each of the following areas: (1) bilateral temporal lobe (temporal superior, middle, inferior, amygdala, hippocampus and parahippocampus, fusiform gyrus, and Heschl gyrus); (2) posterior fossa (vermis, bilateral cerebellum crus, and bilateral cerebellum); (3) bilateral insula; (4) cingulate gyri (anterior cingulate gyrus, middle cingulate gyrus, and posterior cingulate gyrus); (5) bilateral frontal lobe (precentral gyrus, Rolandic operculum, supplementary motor area, olfactory cortex, superior frontal gyrus, middle frontal gyrus, inferior frontal gyrus, gyrus rectus, and paracentral lobule); (6) bilateral occipital lobe (calcarine fissure and surrounding cortex, cuneus, lingual gyrus, and lateral remainder of occipital lobe); (7) bilateral parietal lobe (postcentral gyrus, supramarginal gyrus, angular gyrus, precuneus, parietal, superior, and inferior); (8) bilateral striata (caudate nucleus, putamen, and pallidum); and (9) thalamus.

### Statistical analysis

IBM SPSS was used to compare demographic, clinical, gait, and PET features of PD patients with and without MCI through univariate statistical analysis. First, the normality distribution of the data was assessed with the Shapiro–Wilk and Kolmogorov–Smirnov tests depending on the sample group size (the former for n<50, the latter for n>50). Then, Levene's test was performed for normally distributed data to assess the homoscedasticity of the variances between the groups and, if the statement was verified, a *t*-test for independent samples was employed; otherwise, a Mann–Whitney test was employed.

### Machine learning workflow

The implementation of machine learning algorithms was performed through the KNIME analytics platform (*v. 4.2.1*), which has been used in other biomedical studies with good results (33). In this study, binary supervised classification learning was applied with tree-based and instance-based algorithms. Two models with different subsets of features as input were implemented:

1. Model 1 included age, clinical characteristics (hallucinations/psychosis, depressed mood, anxiety, apathy, sleep problems, FOG), and gait features;
2. Model 2 included quantitative PET features and the top five features of Model 1. Specifically, Model 2A included averaged SUVs of the nine brain areas reported in section 2.4 and the top five features of Model 1, whereas Model 2B included only averaged SUVs of cortical regions and the same top five features of Model 1. Feature importance was computed with RF to identify the most relevant features in classification based on Information Gain (IG). IG is an entropy-based feature evaluation method, which considers how much information a feature can provide and how much this feature can be used in the classification process. In RF, feature importance is estimated by looking at how much prediction error increases when data for a certain variable is permuted while the others are left unchanged. Then, the IG of all the features was normalized and transformed into percentages to express and compare the contribution of each feature to the prediction.

A 10-fold cross-validation was used for the first model, while a leave-one-out technique was used for the second model, considering the sample size. J48, RF, and ADA-B were trained as tree-based algorithms. J48 is an implementation of Quinlan's C4.5 decision tree (34) that uses tree representation to split the information into subsets in which the leaf node corresponds to a class label while the attributes are represented by the branches of the tree. RF is an ensemble learning algorithm that combines the predictions of a high number of decision trees according to the bagging technique, performing randomization (35). ADA-B is another ensemble learning algorithm based on the boosting technique that assigns a group of weights to a set of weak learners and updates it for each iteration to achieve the best performances (36). KNN and SVM were implemented as instance-based algorithms. The former is one of the simplest but most effective classification methods that, starting from a training set with accurate classification labels, define groups of *k* similar samples to a query point in the features space, where similarity can be measured by the distance in the neighborhood (37). The latter is based on finding the optimal separation hyperplane in combination with the maximization of the separating margin (38). Each algorithm has been implemented by tuning the hyperparameters after employing the Optimization Parameter Loop to find the best combinations in terms of score. Particularly, for the SVM algorithm, no weighted matrix of costs was used and a linear kernel type was employed, for the RF algorithm, the number of trees was set to 100 and the split criterion was based on Information Gain Ratio, while for KNN algorithm, *k* was set to 3 and neighbors were weighted by 1-distance, where Euclidean distance was considered.

Finally, algorithms performances were evaluated in terms of accuracy, sensitivity, specificity, and Area Under the Curve Receiver Operating Characteristics (AUCROC).

## Results

### Comparison of demographic, clinical, gait, and amyloid PET features

The comparison of demographic and clinical features between PD-MCI and noPD-MCI patients is provided in Table 1. The two groups did not significantly differ on demographic and anthropometric variables except for age. When comparing clinical variables, the two groups showed a trend toward significance on the Hoehn and Yahr stage and the MDS-UPDRS Depressed Mood item. The comparison of demographic and clinical features between PD-MCI and noPD-MCI of the subgroup of patients undergoing amyloid PET imaging showed similar results (Supplementary Table 1), confirming the homogeneity between the subgroup and the whole group.

**Table 1:** Comparison of demographic and clinical features between PD-MCI and noPD-MCI.

**Features**	**PD-MCI** **(*N* = 33)**	**noPD-MCI** **(*N* = 42)**	***p*** **-value**
Most affected side (R/L)	20/13	23/19	0.611
Body Mass Index	28.32 ± 3.94	27.00 ± 3.21	0.217
Age	65.88 ± 9.06	61.40 ± 8.26	**0.029**
Disease Duration (y)	5.18 ± 2.49	4.56 ± 2.64	0.223
Hoehn & Yahr	1.92 ± 0.31	1.77 ± 0.40	0.081
LEDD (mg)	549.65 ± 382.69	570.49 ± 443.51	0.749
MDS-UPDRS: Part I	9.51 ± 7.50	7.29 ± 4.58	0.332
MDS-UPDRS: Part II	8.00 ± 5.78	8.24 ± 6.07	0.979
MDS-UPDRS: Part III	24.88 ± 8.52	21.67 ± 8.03	0.098
MDS-UPDRS: Part IV	1.52 ± 2.85	2.05 ± 3.09	0.250
MDS-UPDRS Freezing Item (Y/N)	6/27	8/34	0.924
MDS-UPDRS Hallucinations and Psychosis Item	0.14 ± 0.44	0.19 ± 0.37	0.472
MDS-UPDRS Depressed mood Item	0.94 ± 1.20	0.43 ± 0.59	0.085
MDS-UPDRS Anxiety Item	1.03 ± 1.21	0.62 ± 0.80	0.176
MDS-UPDRS Apathy Item	0.64 ± 1.14	0.36 ± 0.79	0.398
MDS-UPDRS Sleep problems Item	0.94 ± 1.09	1.17 ± 1.08	0.311

Statistically significant differences are reported in bold.

MCI, mild cognitive impairment; R/L, right/left; LEDD, levodopa equivalent daily dose; MDS-UPDRS, movement disorders society-unified Parkinson's disease rating scale.

The comparison of gait spatial and temporal parameters during the COG task between PD-MCI and noPD-MCI is reported in Table 2. PD-MCI as compared to noPD-MCI patients showed worse gait parameters. In detail, PD-MCI exhibited increased values of swing duration variability, stance phase, double support phase, and step length variability. Furthermore, PD-MCI patients exhibited reduced mean velocity (both in meters per second and in the percentage of height per second), step length, and cycle length (both in meters and in the percentage of height).

**Table 2:** Comparison of spatial and temporal gait parameters during the COG task between PD-MCI and noPD-MCI patients.

**Features**	**Measurement**	**PD-MCI** **(*N* = 33)**	**noPD-MCI** **(*N* = 42)**	***p*** **-value**
Cycle duration	s	1.18 ± 0.15	1.15 ± 0.13	0.317
Stance duration	s	0.74 ± 0.10	0.70 ± 0.09	0.131
Swing duration	s	0.44 ± 0.06	0.44 ±0.05	0.769
Swing duration variability	s	0.05 ± 0.05	0.03 ± 0.02	**0.015**
Stance phase	%	62.64 ± 2.14	61.40 ± 2.11	**0.010**
Swing phase	%	38.55 ± 5.19	38.54 ± 2.12	0.132
Single support phase	%	37.75 ± 2.16	38.25 ± 2.84	0.401
Double support phase	%	13.88 ± 4.04	11.77 ± 1.89	**0.006**
Mean velocity	m/s	0.81 ± 0.18	0.94 ± 0.17	**0.002**
Mean velocity	%height/s	49.40 ± 11.03	56.24 ± 10.23	**0.005**
Cadence	steps/min	103.85 ± 13.40	106.24 ± 12.42	0.426
Cycle length	m	0.94 ± 0.18	1.06 ± 0.15	**0.003**
Cycle length	%height	57.48 ± 12.78	63.37 ± 8.30	**0.018**
Step length	m	0.42 ± 0.11	0.51 ± 0.10	**< 0.001**
Step length variability	m	0.33 ± 0.48	0.11 ± 0.24	**0.002**
Step width	m	0.11 ± 0.07	0.12 ± 0.11	0.797

Statistically significant differences are reported in bold.

COG task, cognitive dual task; MCI, mild cognitive impairment.

A subgroup of PD patients, namely 36 subjects, including 19 PD-MCI and 17 noPD-MCI, underwent amyloid PET imaging. As reported in Table 3, when comparing PD-MCI vs. noPD-MCI, the two groups did not significantly differ on amyloid PET tracer retention for all brain areas under examination.

**Table 3:** Comparison of averaged SUVs evaluating amyloid PET tracer retention in brain areas in PD-MCI and noPD-MCI patients.

**Side**	**Brain area**	**PD-MCI** **(*N* = 19)**	**noPD-MCI** **(*N* = 17)**	***p*** **-value**
Right	Frontal	1.04 ± 0.42	0.91 ± 0.25	0.379
	Parietal	1.05 ± 0.46	0.90 ± 0.24	0.379
	Temporal	0.99 ± 0.42	0.91 ± 0.24	0.661
	Occipital	1.05 ± 0.45	0.93 ± 0.23	0.552
	Insula	0.92 ± 0.49	0.94 ± 0.39	0.950
	Striatum	1.06 ± 0.43	0.99 ± 0.28	0.778
	Cingulum	1.13 ± 0.53	1.05 ± 0.28	0.975
	Thalamus	1.11 ± 0.49	1.04 ± 0.30	0.925
	Posterior fossa	0.82 ± 0.27	0.77 ± 0.22	0.594
Left	Frontal	1.03 ± 0.40	0.92 ± 0.23	0.433
	Parietal	1.05 ± 0.47	0.94 ± 0.25	0.531
	Temporal	0.99 ± 0.40	0.89 ± 0.24	0.531
	Occipital	1.00 ± 0.46	0.93 ± 0.22	0.827
	Insula	1.06 ± 0.47	0.89 ± 0.29	0.433
	Striatum	1.07 ± 0.36	0.97 ± 0.32	0.552
	Cingulum	1.06 ± 0.44	1.01 ± 0.35	0.925
	Thalamus	1.20 ± 0.43	1.07 ± 0.38	0.300
	Posterior fossa	0.87 ± 0.38	0.79 ± 0.22	0.707

SUV, standardized uptake values; PET, positron emission tomography; MCI, mild cognitive impairment.

### Machine learning analysis

The evaluation metrics per each model and each algorithm are summarized in Table 4.

**Table 4:** Evaluation metrics per each algorithm and per each model, where Model 1 included clinical and gait features during the COG task, Model 2 included PET features (Model 2A included cortical and subcortical regions, Model 2B included only cortical regions), and the top five features of Model 1.

**Features**	**Algorithms**	**Accuracy**	**Sensitivity**	**Specificity**	**AUCROC**
Model 1 (*n* = 75)	SVM	80.0	72.7	85.7	0.792
	KNN	70.7	66.7	73.8	0.696
	J48	73.3	66.7	78.6	0.639
	ADA-B	70.7	57.6	81.0	0.685
	RF	73.3	66.7	78.6	0.722
Model 2A (*n* = 36)	SVM	72.2	73.7	70.6	0.721
	KNN	66.7	52.6	82.4	0.567
	J48	72.2	73.7	70.6	0.621
	ADA-B	66.7	73.7	58.8	0.625
	RF	63.9	73.7	52.9	0.573
Model 2B (*n* = 36)	SVM	75.0	73.7	76.5	0.751
	KNN	63.9	57.9	70.6	0.639
	J48	69.4	68.4	70.6	0.636
	ADA-B	63.9	57.9	70.6	0.693
	RF	61.1	63.2	58.8	0.577

SVM, Support vector machine; KNN, k-nearest neighbor; J48, decision tree; ADA-B, adaBoost; RF, random forest.

Regarding Model 1, employing clinical and both spatial and temporal gait variables during the COG task, SVM obtained the best results in terms of accuracy (80.0%), sensitivity (72.7%), specificity (85.7%), and ACUROC (79.2%), followed by RF in terms of accuracy (73.3%), specificity (78.6%), and AUCROC (72.2%).

Otherwise, regarding Model 2A, including Averaged SUVs evaluating amyloid PET tracer retention of all brain areas reported in section 2.4 and the top five features of Model 1 (step length, cycle length-%height, step length variability, mean velocity, and stance phase), all the algorithms got worse results than Model 1. Indeed, again the highest results were obtained by SVM (accuracy = 72.2%, sensitivity = 73.7%, specificity = 70.6%, AUCROC = 72.1%). J48 also obtained good results in terms of accuracy (72.2%), sensitivity (73.7%), and specificity (70.6%), while KNN got the worst results in terms of sensitivity (52.6%) and AUCROC (56.7%). When including in the model only cortical amyloid PET tracer retention and the top five features of Model 1 (Model 2B), evaluation metrics were quite similar to Model 2A, as shown in Table 4.

Below, there is the feature importance of the top five features chosen from Model 1:

- Step length (m), 9.15%:
- Cycle length (%height), 7.54%;
- Step length variability (m), 7.38%;
- Mean velocity (m/s), 7.28%;
- Stance phase, 7.14%.

**Figure 1:**
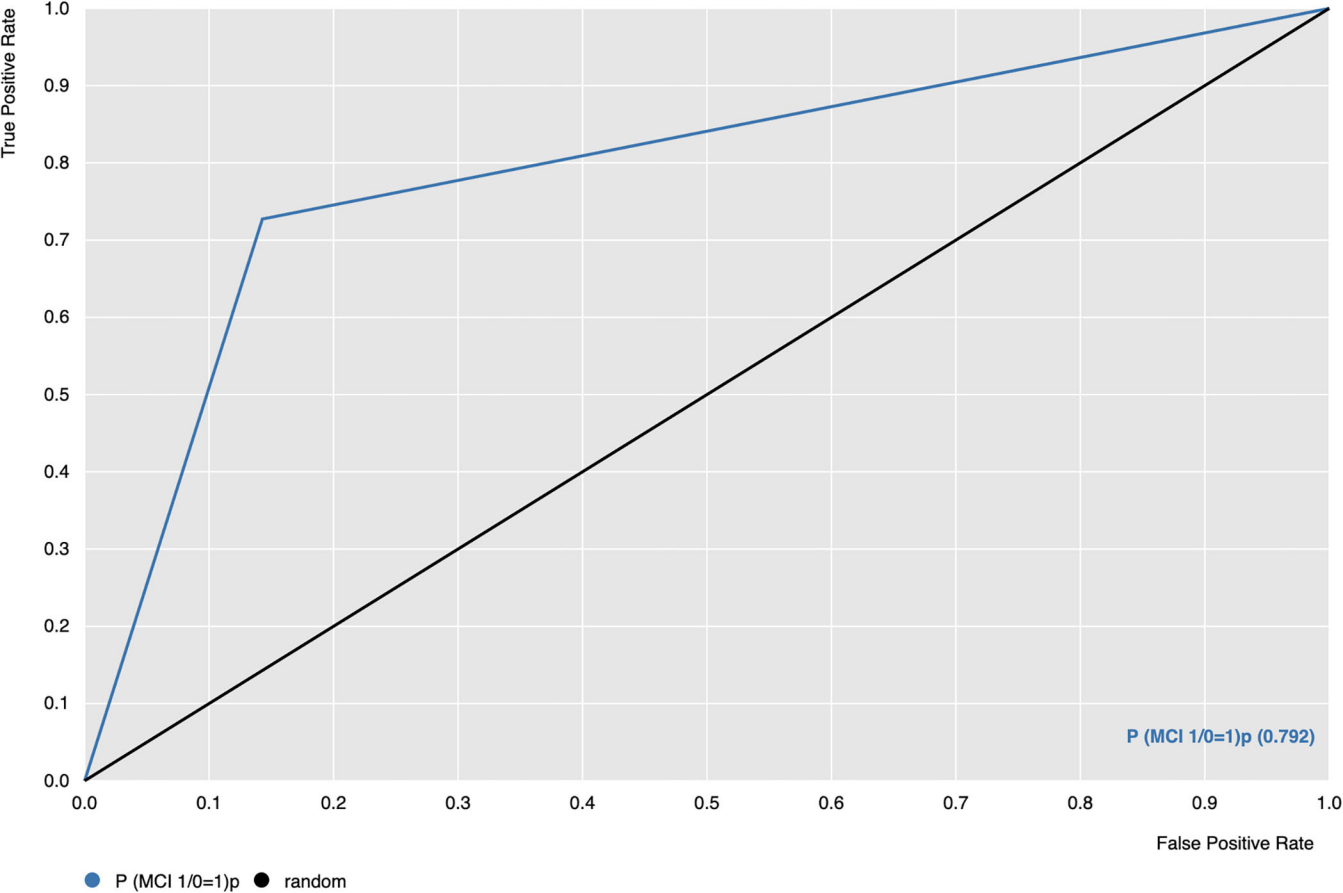
Receiver Operating Characteristic (ROC) curve of SVM algorithm (blue line); ROC = 0.5, threshold for considering the model better than random guessing (black line). P(MCI 1/0 = 1) (orange line) is the probability of the model correctly classifying group 1, which corresponds to PD-MCI patients.

Figure 1 shows the Receiver Operating Characteristic (ROC) curve of the SVM algorithm of Model 1, indicating that the model is capable of distinguishing and correctly classifying the two groups better than random guessing with a probability *p* = 0.792.

**Figure 2:**
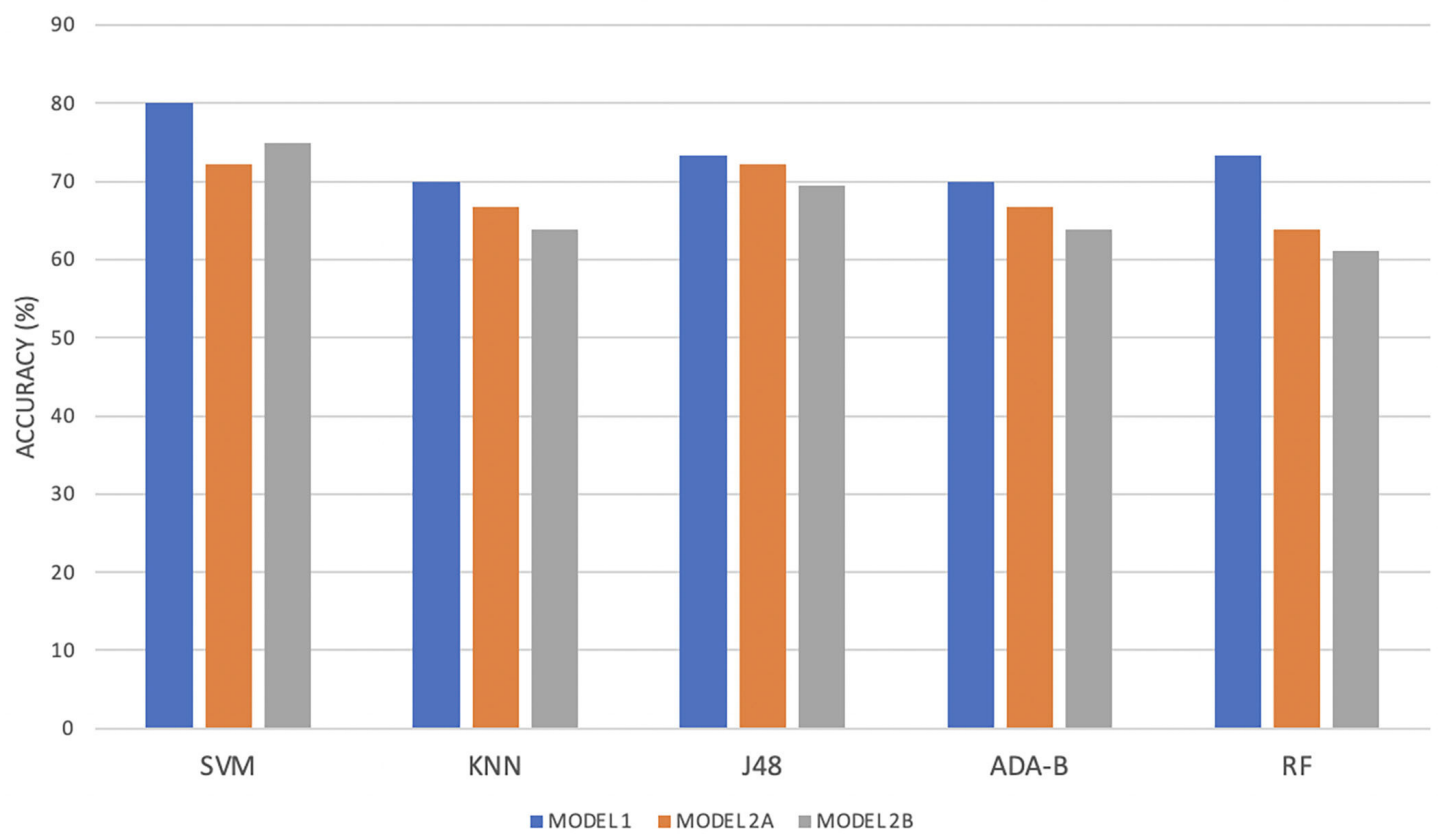
Level of agreement among classifiers' scores for each model of the analysis.

Finally, Figure 2 shows the level of agreement among classifiers' scores and allows us to compare the best results for each model of the analysis. It highlights that SVM is capable of correctly classifying the two groups for all three models.

## Discussion

To our knowledge, this is the first study exploring the respective burden of different features commonly associated with PD-MCI by using machine learning approaches with the main aim of identifying the strongest indicators of PD-MCI and, as a consequence, detecting a machine learning algorithm able to distinguish PD patients with MCI from those without. For this purpose, clinical features, gait variables under dual-task conditions, and brain β-amyloid load were first compared between noPD-MCI and PD-MCI patients using univariate statistical analysis and then employed to implement two multiparametric machine learning models.

When comparing demographic and clinical features between noPD-MCI and PD-MCI patients, the latter showed significantly older age and a trend on both more advanced stage and increased depressive symptoms than the first, consistently with previous findings (1, 39).

Following previous studies (12–15, 40), during the dual task, PD-MCI, as compared to noPD-MCI patients, showed reduced velocity, a raw measure underlain by multiple gait adaptations, and exhibited dysfunctions on spatial gait variables, namely reduced step length and, likely as a consequence, cycle length. In addition, PD-MCI showed a longer stance phase, mostly in double support, and increased measures of gait variability, that is, swing duration variability and step length variability, which are markers of dynamic unbalance (41). These findings further corroborate the notion that dual-task conditions exert detrimental effects on dynamic stability, especially in patients with cognitive decline (16, 42, 43) with consequent increased risk of falling. Importantly, since all patients were on medication during gait analysis, the increased gait dysfunctions observed in PD-MCI vs. noPD-MCI might reflect their levodopa-resistant nature, thus supporting widespread neurotransmitters deficits beyond dopamine in PD patients with cognitive decline [for a recent review, see (6)].

While comparing β-amyloid load in the subgroup of PD-MCI vs. noPD-MCI, we failed to find any significant difference in all examined cortical and subcortical areas. Indeed, our findings are consistent with recent research that equally did not support an association between increased Aβ deposition and cognitive decline in PD (17, 18, 44). It is worth noting that we compared the two groups on averaged SUVs evaluating β-amyloid load as continuous variables, being the main aim of the present study to use amyloid PET measures as continuous features implementing machine learning models.

Previous recent studies focused on MCI in PD patients through machine learning approaches. Abós et al. (45) employed fMRI images to implement machine learning algorithms to predict and classify PD patients with and without MCI, achieving an accuracy of 82.6%.

Meanwhile, Tsiouris et al. (46) employed many heterogeneous baseline variables (clinical features, MRI/DatScan-SPECT imaging, laboratory results, and genetic analysis) from newly diagnosed patients of the Parkinson's Progression Markers Initiative cohort to identify risk factors for early cognitive impairment using machine learning techniques. They found that older age, cognitive dysfunction, sleep problems, daytime sleepiness, smell dysfunction, mood impairment, and anxiety represent strong determinants of the development of early MCI or dementia in the first 5 years of PD with an accuracy of 80.38%.

Differently, Chen et al. (47) implemented machine learning models, using gait analysis parameters obtained from a portable system, to distinguish PD-MCI patients from subjects with MCI without PD, reaching really good results in terms of accuracy (91.67%) and AUCROC (97.14%). The excellent accuracy of these models may be mirrored by walking differences between subjects with and without PD rather than gait differences specifically related to the type of MCI.

Finally, in previous research, we used quantitative gait features in both single and dual task to train algorithms with a previous data-augmentation technique, due to small sample size, and obtained good results in terms of accuracy (86.8%), sensitivity (88.2%), and AUCROC (90.0%) in differentiating PD patients with and without MCI (16).

In comparison with these previous researches, in the present study, we implemented machine learning techniques with multiparametric features that are commonly associated with cognitive decline in PD but stemming from different sources, namely selected clinical features, gait parameters under dual-task condition, and β-amyloid load in cortical and subcortical regions, to identify possible hidden patterns underpinning MCI. When implementing the first model including age, clinical features, and gait variables under dual task, we found that it was able to distinguish PD-MCI from noPD-MCI with good accuracy (80.0%), sensitivity (72.7%), specificity (85.7%), and AUCROC (79.2%). Interestingly, the top five features chosen from Model 1 included only gait parameters, namely step length, cycle length, step length variability, velocity, and stance phase, thus corroborating that quantitative gait measures, especially under dual-task conditions, may represent a reliable surrogate biomarker of cognitive decline in PD (13, 14, 48). It is worth noting that the gait parameters selected by Model 1 could also be obtained by using a quick assessment like wearable sensors, thus making it easy to apply our outcomes. Meanwhile, Model 2A, which included cortical and subcortical amyloid PET tracer retention values and the top five features of Model 1, achieved worse evaluation metrics (accuracy = 72.2%, sensitivity = 73.7%, specificity = 70.6%, AUCROC = 72.1%), thereby excluding that amyloid deposition might predict the presence of MCI in PD, even when ruling out subcortical amyloid PET tracer retention (Model 2B). Of note, we included in our model the β-amyloid uptake ratio, indeed exploring whether even low β-amyloid deposition, so below AD-range thresholds, may play a role in the presence of an ongoing multisystem neurodegenerative process like PD. Taken together, on the one hand, our findings further demonstrate the close relationship between specific gait parameters and MCI in PD, and on the other, they do not support that β-amyloid deposition might have a prominent role in the pathogenesis of MCI in PD.

From a speculative perspective, our results suggest that peculiar gait alterations and cognitive decline are may be underpinned by dysfunctions on common neural networks including non-motor cortical and subcortical areas and non-dopaminergic networks (49–52). It is worth noting that discrete cognitive components, notably step length, step length variability, and stance phase during medication and under dual task, show specific association with MCI as demonstrated by univariate statistical analysis and further confirmed by machine learning implementation. Given that gait dysfunctions may antedate cognitive decline in PD (13, 53), it is conceivable that these gait measures might serve as early markers of future cognitive decline. Conversely, clinical features and amyloid deposition do not seem to represent reliable markers of early cognitive dysfunction. The lack of association in our study between clinical features, such as hallucinations/psychosis, apathy, sleep disorders, anxiety, depression and FOG, and MCI may be due to several reasons: (1) the reduced reliability of MDS-UPDRS on measuring such symptoms; (2) the prominent association of these features with more advanced cognitive decline, that is, PDD (6); (3) the combination of both previous explanations. Finally, our machine learning findings indicate that the expression of walking abnormalities associated with early cognitive decline is likely not mediated by β-amyloid pathology, given that Model 2 (both A and B) as compared with Model 1 exhibits even worse evaluation metrics in MCI prediction.

The present study has some limitations. First, the small sample size of amyloid PET data could have influenced algorithms predictions of Model 2, thus assuming that the present findings should be confirmed with larger samples. Second, we did not include an age-matched control group in our analysis, nonetheless, a direct comparison with a control group was beyond the purpose of the present study. Third, the selection criteria of our study could have contributed to excluding a subpopulation of PD patients with worse progression, namely early dementia and/or disabling gait dysfunction, not allowing generalizing our conclusions to PD patients with the malignant course. Finally, we rated clinical non-motor features and FOG with the respective MDS-UPDRS items, thereby using a five-point scale for each symptom that could have affected the reliability of the feature quantification. In addition, though MDS-UPDRS items may represent strength for screening mental non-motor symptoms and building an easy algorithm, their accuracy in capturing the presence and severity of such symptoms is limited.

In conclusion, despite these limitations, here, we demonstrate that a data mining approach using specific clinical and quantitative gait variables exhibits high accuracy, specificity, and sensitivity in predicting the presence of MCI in PD, whereas gray matter amyloid PET tracer retention does not appear to increase prediction. In addition, our results provide a proof of concept that machine learning techniques implemented with certain gait parameters might represent a reliable surrogate biomarker of cognitive impairment in PD.

## Data availability statement

The raw data supporting the conclusions of this article will be made available by the authors, without undue reservation.

## Ethics statement

The studies involving human participants were reviewed and approved by the IRCCS G. Pascale Foundation, the reference Ethics Committee of the IRCCS SDN SYNLAB. The patients/participants provided their written informed consent to participate in this study.

## Author contributions

MA, CR, SA, EN, RE, LP, and PB contributed to conception and design of the study. AV, SC, GC, and LB organized the database. CR, SA, and GD'A performed the statistical analysis. MA, CR, and SA wrote the first draft of the manuscript. EN, LB, and MS wrote sections of the manuscript. All authors contributed to manuscript revision, read, and approved the submitted version.

## Funding

The present study has been funded by the University of Salerno (FARB2020).

## Conflict of interest

Unrelated to this study, PB received consultancies as a member of the advisory board for Zambon, Lundbeck, UCB, Chiesi, Abbvie, and Acorda; RE received consultancies from Zambon and honoraria from TEVA. The remaining authors declare that the research was conducted in the absence of any commercial or financial relationships that could be construed as a potential conflict of interest.

## Publisher's note

All claims expressed in this article are solely those of the authors and do not necessarily represent those of their affiliated organizations, or those of the publisher, the editors and the reviewers. Any product that may be evaluated in this article, or claim that may be made by its manufacturer, is not guaranteed or endorsed by the publisher.
