# Supplementary material for: Machine learning can predict mild cognitive impairment in Parkinson's disease
Source: Front Neurol. 2022 Nov 17;13:1010147. doi: 10.3389/fneur.2022.1010147 (PMC9714435; doi:10.3389/fneur.2022.1010147)
Supplement: Supplementary file 1 [file Table_1.DOCX]

**Table S1. Comparison of demographic and clinical features between PD-MCI and noPD-MCI of the subgroup of PD patients**

| **Features** | **PD-MCI**  **(N=19)** | **noPD-MCI**  **(N=17)** | **p-value** |
| --- | --- | --- | --- |
| Most affected side (R/L) | 12/7 | 9/8 | 0,535 |
| Body Mass Index | 28,47 ± 4,01 | 28,53 ± 2,46 | 0,954 |
| Age | 63,58 ± 7,19 | 61,00 ± 6,26 | 0,262 |
| Disease Duration (y) | 5,47 ± 2,52 | 5,52 ± 2,62 | 0,958 |
| Hoehn & Yahr | 1,87 ± 0,40 | 1,71 ± 0,44 | 0,219 |
| LEDD (mg) | 523,66 ± 348,60 | 671,15 ± 442,49 | 0,375 |
| MDS-UPDRS: Part I | 10,53 ± 8,02 | 7,06 ± 4,34 | 0,226 |
| MDS-UPDRS: Part II | 9,05 ± 6,29 | 8,65 ± 5,14 | 0,762 |
| MDS-UPDRS: Part III | 25,11 ± 9,56 | 21,24 ± 7,80 | 0,195 |
| MDS-UPDRS: Part IV | 1,32 ± 2,65 | 2,65 ± 3,62 | 0,210 |
| MDS-UPDRS Freezing Item (Y/N) | 4/15 | 5/12 | 0,563 |
| MDS-UPDRS Hallucinations and Psychosis Item | 0,21 ± 0,54 | 0,06 ± 0,24 | 0,338 |
| MDS-UPDRS Depressed mood Item | 1,11 ± 1,20 | 0,41 ± 0,51 | 0,066 |
| MDS-UPDRS Anxiety Item | 1,05 ± 1,39 | 0,47 ± 0,62 | 0,301 |
| MDS-UPDRS Apathy Item | 0,89 ± 1,29 | 0,24 ± 0,56 | 0,083 |
| MDS-UPDRS Sleep problems Item | 0,84 ± 1,12 | 1,35 ± 1,06 | 0,107 |

MCI: Mild Cognitive Impairment; R/L: Right/Left; LEDD: Levodopa Equivalent Daily Dose; MDS-UPDRS: Movement Disorders Society-Unified Parkinson Disease Rating Scale.
